# Exploring the therapeutic mechanisms of Yikang decoction in polycystic ovary syndrome: an integration of GEO datasets, network pharmacology, and molecular dynamics simulations

**DOI:** 10.3389/fmed.2024.1455964

**Published:** 2024-10-03

**Authors:** Jiang Miao, LiXuan Gao, Xi Liu, Wenpin Cai, Lei Chen, Mojinzi Chen, Yun Sun

**Affiliations:** ^1^Department of Pharmacy, Wenzhou TCM Hospital of Zhejiang Chinese Medical University, Wenzhou, China; ^2^Department of Rehabilitation Medicine, Wenzhou TCM Hospital of Zhejiang Chinese Medical University, Wenzhou, China; ^3^Wuyanling National Natural Reserve Administrative of Zhejiang, Wenzhou, China; ^4^Department of Laboratory Medicine, Wenzhou TCM Hospital of Zhejiang Chinese Medical University, Wenzhou, China; ^5^Department of Chinese Internal Medicine, Wenzhou Integrated Traditional Chinese and Western Medicine Hospital of Zhejiang Chinese Medical University, Wenzhou, China; ^6^Department of Gynaecology, Wenzhou TCM Hospital of Zhejiang Chinese Medical University, Wenzhou, China

**Keywords:** Yikang decoction, polycystic ovary syndrome, network pharmacology, molecular docking, molecular dynamics simulation

## Abstract

**Objective:**

The incidence of Polycystic Ovary Syndrome (PCOS) is increasing annually. This study aims to investigate the therapeutic mechanisms of Yikang Decoction (YKD) in the treatment of PCOS through the integration of GEO datasets, network pharmacology, and dynamic simulation.

**Methods:**

Active ingredients of YKD and their targets were collected from the Traditional Chinese Medicine Systems Pharmacology (TCMSP) platform. Disease-relevant targets for PCOS were retrieved from several databases, including GeneCards, OMIM, PharmGKB, DrugBank, and GEO. The underlying pathways associated with the overlapping targets between YKD and PCOS were identified using Gene Ontology (GO) and Kyoto Encyclopedia of Genes and Genomes (KEGG) enrichment analysis. The mechanisms of interaction between the core targets and components were further explored through molecular docking and molecular dynamics simulations (MD).

**Results:**

139 potential active components and 315 targets of YKD were identified. A topological analysis of the PPI network revealed 10 core targets. These targets primarily participated in the regulation of biological processes, including cell metabolism, apoptosis, and cell proliferation. The pathways associated with treating PCOS encompassed PI3K-Akt signaling pathway, Lipid and atherosclerosis, MAPK signaling pathways, and Endocrine resistance signaling pathways. Moreover, molecular docking and MD have been shown to reveal a good binding capacity between active compounds and screening targets.

**Conclusion:**

This study systematically investigates the multi-target mechanisms of YKD in the treatment of PCOS, with preliminary verification provided through molecular docking and MD. The findings offer compelling evidence supporting the efficacy of YKD in treating PCOS.

## 1 Introduction

Polycystic ovary syndrome (PCOS) is a common condition affecting women of reproductive age. It is characterized by a range of abnormalities that impact multiple systems, including the reproductive, metabolic, and psychological components, throughout the entire life cycle (1, 2). Epidemiologic surveys estimate that the global prevalence of PCOS is approximately 8–13% (3). In China, the prevalence is about 5.6% and shows a rising trend annually (4). The clinical manifestations of PCOS are highly variable, but are primarily marked by hyperandrogenism, polycystic ovarian changes, and irregular menstruation or amenorrhea. These manifestations are frequently accompanied by varying degrees of insulin resistance (IR), metabolic issues such as obesity, and mood disorders, including anxiety and depression (5, 6). The symptoms of PCOS not only cause physical and psychological distress for patients but also present significant challenges to their fertility. Moreover, they impose a considerable burden on families and society.

Currently, the management of PCOS primarily emphasizes symptomatic relief through exercise, dietary changes, ovulation-inducing medications, and assisted reproductive technologies (7). Despite these efforts, challenges remain, including extended treatment durations, multiple complications, and less-than-ideal therapeutic outcomes (8). Research has shown that oral contraceptives can have adverse effects such as hormone disruption, vertigo, migraines, and irregular blood pressure and blood sugar levels (9). Moreover, metformin, often prescribed for PCOS, may cause gastrointestinal issues including abdominal pain, diarrhea, nausea, and vomiting (10). It may also result in elevated aminotransferase levels, which can indicate potential liver function problems. Long-term use of these medications can lead to endocrine disruption, osteoporosis, and liver dysfunction, as well as emotional and mental health issues that may negatively impact patient adherence (11). Consequently, research into PCOS is crucial for its medical, economic, and social implications, as it plays a significant role in improving patient well-being and advancing societal progress.

Traditional Chinese Medicine (TCM) offers distinct advantages in the treatment of PCOS, including holistic management, evidence-based approaches, safe medications, and individualized treatment plans (12, 13). Yikang Decoction (YKD) is an empirical formula created by Sun Yun, a renowned Ouyue doctor from Wenzhou City, China. The formula comprises 13 herbs: Radix Lithospermi (Zicao, ZC), Radix Bupleuri (Chaihu, CH), Radix Rubiae (Qiancao, QC), Radix Salviae liguliobae (Danshen, DS), Cortex Moutan (Mudanpi, MDP), Folium Eriobotryae (Pipaye, PPY), Gentiana scabra var. buesgeri (Longdan, LD), Radix Cyathulae (Chuanniuxi, CNX), Rhizoma Cyperi Preparata (Cuxiangfu, CXF), Rhizoma Dioscoreae (Shanyao, SY), Fructus Gardeniae (Zhizi, ZZ), Radix Puerariae (Gegen, GG), and Semen Cuscutae (Tusizi, TSZ). This formulation is known for its effects of clearing liver fire, activating blood circulation, removing blood stasis, and dredging the veins. Our research group has found that YKD can effectively improve patients’ hormone levels and clinical symptoms (14, 15). However, the precise biomolecular pharmacological effects of this compound remain unknown due to its complex chemical nature.

In recent years, the rapid advancement of bioinformatics has introduced significant ideas for systematically studying the mechanisms of action in TCM compounding, garnering widespread attention and application. Network pharmacology can be employed in researching TCM compounding to reveal the interactions among various drug components and their associations with targets in the human body, thereby contributing to the comprehensive understanding of the mechanisms of action of Chinese medicines (16). This approach aligns perfectly with the principle of holistic treatment in TCM, highlighting the complex nature of multi-component, multi-target, and multi-pathway interactions inherent in compound prescriptions. Molecular docking technology facilitates the analysis of interactions and binding between active components and target proteins, aiding in the understanding of their effectiveness and potential mechanisms of action (17). Similarly, MD simulations can model binding interactions and study the movement behavior and structural changes of active ingredients and target proteins over different time scales, further elucidating the pharmacological actions and properties of these ingredients (18). This study utilizes a network pharmacology approach to elucidate the mechanism of action of YKD on PCOS. It is validated through molecular docking and MD simulations, offering new research directions and insights for the clinical treatment of PCOS.

## 2 Materials and methods

**Figure 1:**
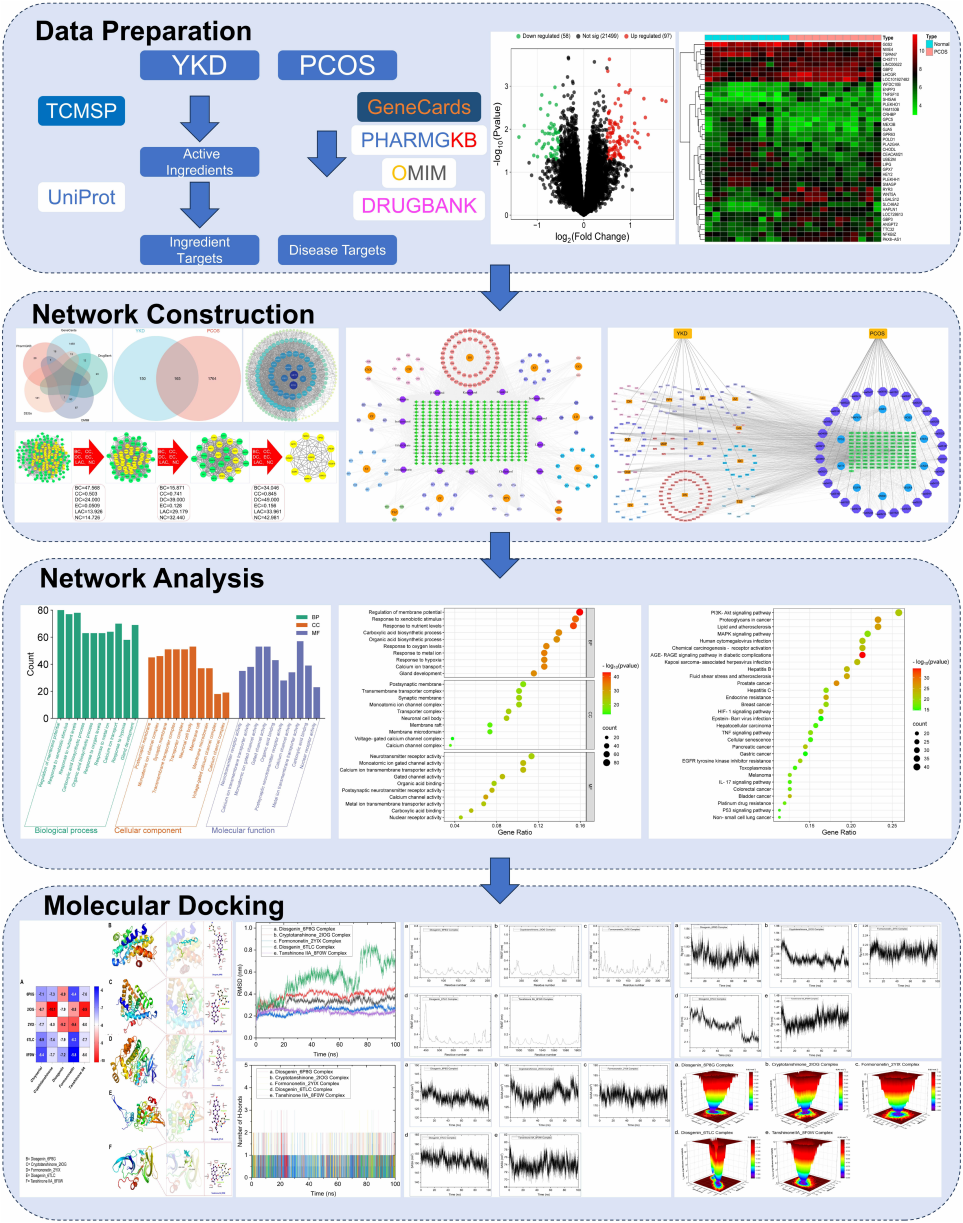
Flow chart of the study strategy.

Figure 1 illustrates the process employed in this research study.

### 2.1 Data download and organization from GEO

The gene expression profile dataset GSE80432 was retrieved from the GEO database, which is affiliated with the National Center for Biotechnology Information. This dataset, obtained using ‘Polycystic Ovary Syndrome’ as the search keyword, includes gene chip data from a total of 16 samples.

### 2.2 Data analysis using Perl and R languages

Background correction and matrix data normalization were carried out based on the characteristics of the data samples. Gene differential analysis of the microarray data was performed using the limma R package. The filtering criteria for identifying significantly different genes were set with a *p*-value threshold of <0.05, |logFC| ≥ 0.5 (FC is fold change), and the differentially expressed genes (DEGs) were identified. The ggplot2 package was applied to create a volcano plot of the microarray data, while the pheatmap package was used to draw the heat map of the gene chip.

### 2.3 Active ingredients and targets prediction

Leveraging the TCMSP database,^1^ effective components and their target proteins were identified by applying the criteria of Oral Bioavailability (OB) ≥ 30% and Drug-Likeness (DL) ≥ 0.18.

### 2.4 Construction of the “YKD-active ingredients-targets of action” network diagram

The active ingredients and targets information were organized in Excel to create the network and type files. This data was subsequently imported into Cytoscape 3.10.1 to construct the ‘YKD-active ingredients - targets network and analyze its properties.

### 2.5 Collection of PCOS-related targets

Keyword searches for “polycystic ovary syndrome” were conducted across several databases, including GeneCards,^2^ OMIM,^3^ PharmGKB,^4^ and DrugBank.^5^ The resulting data were then consolidated and duplicates were removed. We subsequently integrated these results with differential expression genes from GEO, further eliminating any duplicate entries to create a comprehensive inventory of PCOS.

### 2.6 Mapping YKD-PCOS protein interactions and core targets screening

A comparison was conducted between the target genes associated with YKD and PCOS using the Venn platform, which produced a Venn diagram. The STRING database^6^ was utilized to construct the Protein–Protein Interaction (PPI) network for the intersecting genes, focusing on the “*Homo sapiens*” species with a confidence level set at 0.400. This network was then imported into Cytoscape version 3.10.1. Within Cytoscape, the CytoNCA module was employed to analyze six metrics: Betweenness Centrality (BC), Closeness Centrality (CC), Degree Centrality (DC), Eigenvector Centrality (EC), Local Average Connectivity (LAC), and Network Centrality (NC). Genes that surpassed the median value for these metrics were selected, and this subset was used to construct the core target network.

### 2.7 Enrichment analysis

The overlapping genes of YKD-PCOS were analyzed using GO and KEGG enrichment analyses. These analyses were performed using the “Bioconductor” package in R software and the bioinformatics platform.^7^ The results were visualized through bar charts and bubble charts.

### 2.8 Construction of the YKD active ingredients-intersecting gene-PCOS-KEGG pathways network map

The network map illustrating the interaction between YKD active ingredients and gene-PCOS-KEGG pathways was constructed using Cytoscape 3.10.1. Network properties were analyzed using the “Network Analyzer” module. Genes with scores exceeding the mean were selected as key targets based on degree sorting.

### 2.9 Molecular docking

The mol2 files for the primary active ingredients were sourced from PubChem. Core target proteins were screened using the UniProt^8^ and PDB^9^ databases. Molecular docking was conducted with AutoDock Vina, and the results were visualized using PyMoL. Heat mapping was performed using GraphPad Prism 10.1.2.

### 2.10 Molecular dynamics simulations

Molecular dynamics simulations of the complexes were conducted using the GROMACS 2020.3 software suite, with the Amber 99SB force field and the TIP3P water model. The simulation spanned 100,000 ps. Following docking, hydrogenation of the small molecule ligands was performed using Avogadro 1.2.0. The hydrogenated ligands were then uploaded to the CGenFF website to generate a topology file. This topology file was combined with the protein topology file to create the comprehensive topology files for the system. A dodecahedral box, utilizing the Simple Point Charge (SPC) water model, was used to enclose the complex. The distance between the complex and the boundary of the enclosure was set to 1 nm. Sodium or chloride ions were added to neutralize the system’s charge. After energy minimization, the system was equilibrated at a temperature of 310 K and a pressure of 1 bar. A subsequent simulation lasting 100 ns was performed. The trajectory files were analyzed to determine system parameters such as Root Mean Square Deviation (RMSD), Root Mean Square Fluctuation (RMSF), Radius of Gyration (Rg), Solvent Accessible Surface Area (SASA), and Principal Component Analysis (PCA). The results were visualized using Origin 2021.

## 3 Results

### 3.1 Screening of DEGs

**Figure 2:**
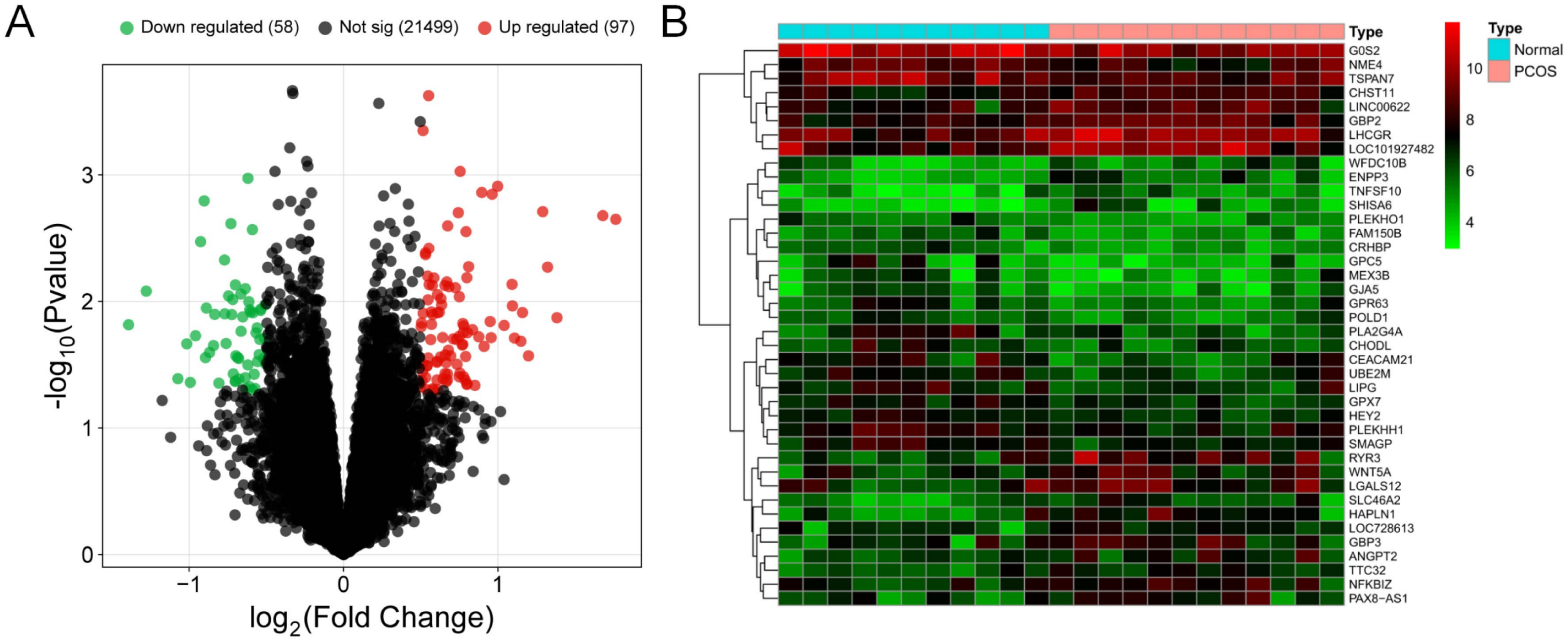
**(A)** Volcano plot and **(B)** Dendrogram for DEGs.

Following data preprocessing, as illustrated in the volcano plot (Figure 2A), we identified 155 differentially expressed genes in PCOS tissues, with 58 genes showing down-regulation and 97 genes showing up-regulation. The top 20 genes with the most significant up-regulation and down-regulation were selected and analyzed (Figure 2B).

### 3.2 Active ingredients and action targets of YKD

**Figure 3:**
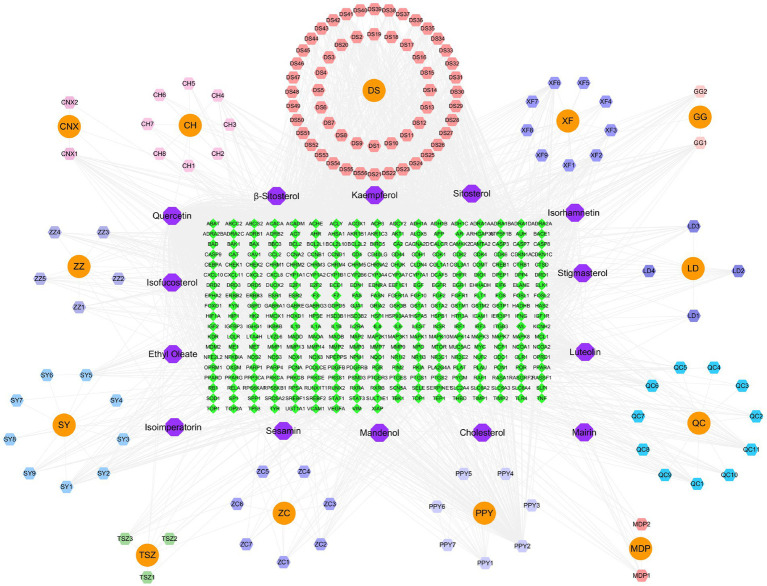
“YKD-ingredient-target” network diagram.

Through a search of the TCMSP database and integration with the UniProt database to convert standard gene symbols, we identified a total of 139 active ingredients. These ingredients were associated with 315 target points of action, underscoring the molecular complexity of the formula and its potential for multiple mechanisms of action. The network diagram illustrating the “active ingredients - targets” relationships, constructed based on these data, is presented in Figure 3.

### 3.3 PCOS disease targets

**Figure 4:**
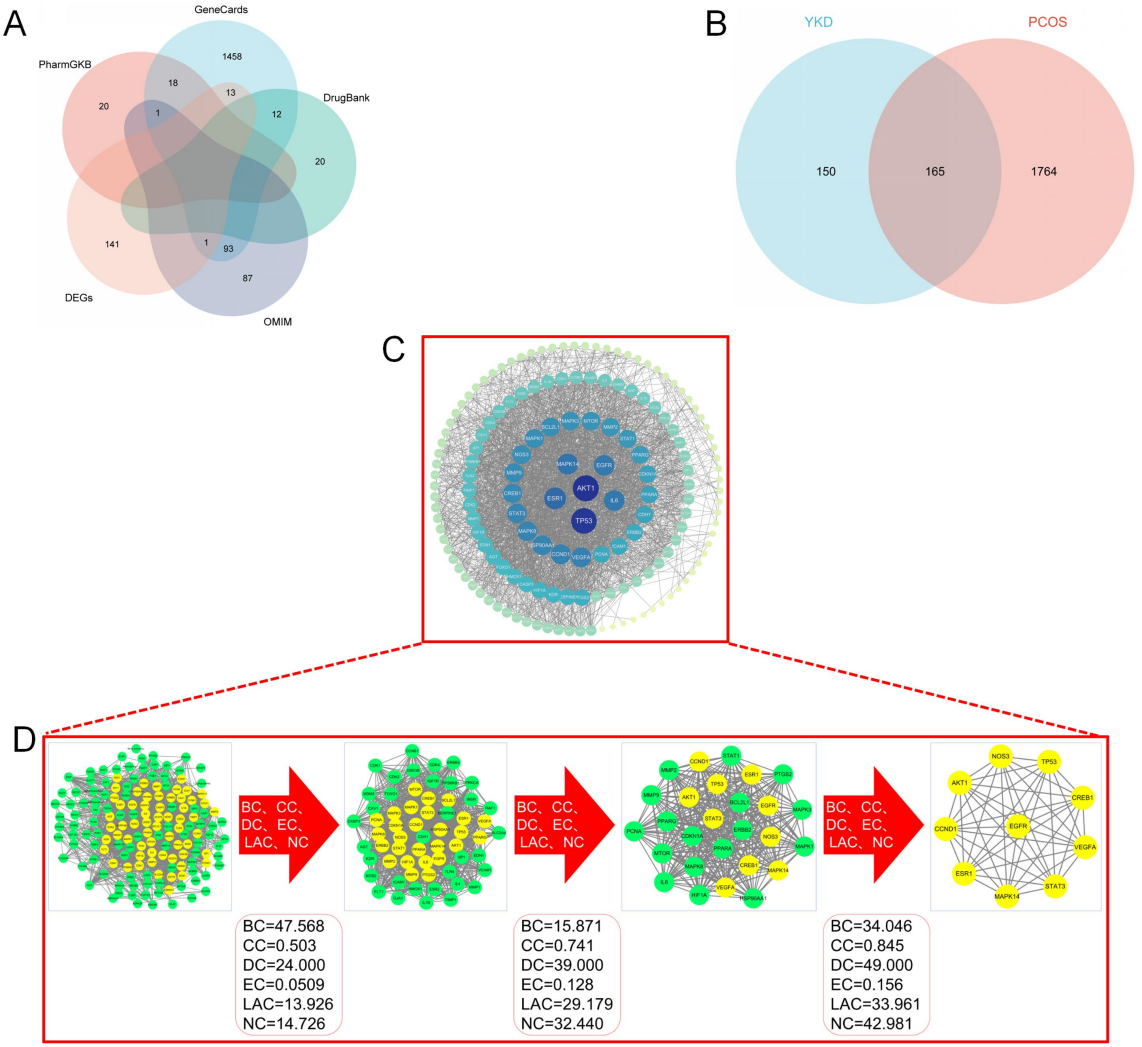
**(A)** Venn diagram of “PCOS” targets; **(B)** Venn diagram of “YKD-PCOS” targets; **(C)** PPI network diagram of “YKD-PCOS” targets; **(D)** Results of key targets screening.

Through systematic mining of GeneCards, OMIM, PharmGKB, DrugBank, and other databases, and after removing duplicates, a total of 1,929 disease targets were identified (Figure 4A). Comparing these targets with those associated with the sexual components of the group formula revealed 165 common genes (Figure 4B).

### 3.4 Constructing a PPI network and identifying crucial targets through screening

The PPI network graph was constructed using the STRING platform, incorporating 165 intersecting genes from YKD and PCOS. A confidence level of 0.400 was specified, and free nodes were eliminated. Online analysis revealed that the network comprised 165 nodes and 1,611 edges, with an enrichment *p*-value of <1.0e-16, an average node degree of 51.9, and an average local clustering coefficient of 0.675. The PPI results were subsequently imported into Cytoscape 3.10.1 for topological analysis, and the network graph was plotted based on the degree values (Figure 4C). The analysis showed that the mean degree centrality of this network is 29.11.

The CytoNCA module employed six indicators BC, CC, DC, EC, LAC, and NC-to identify key genes. After three rounds of optimization, genes with values surpassing the median were selected as key genes. Ultimately, 10 essential genes were identified: TP53, VEGFA, MAPK14, CREB1, ESR1, STAT3, CCND1, AKT1, NOS3, and EGFR. These genes represent 6.06% of the intersecting genes, as shown in Figure 4D.

### 3.5 GO and KEGG enrichment analysis

**Figure 5:**
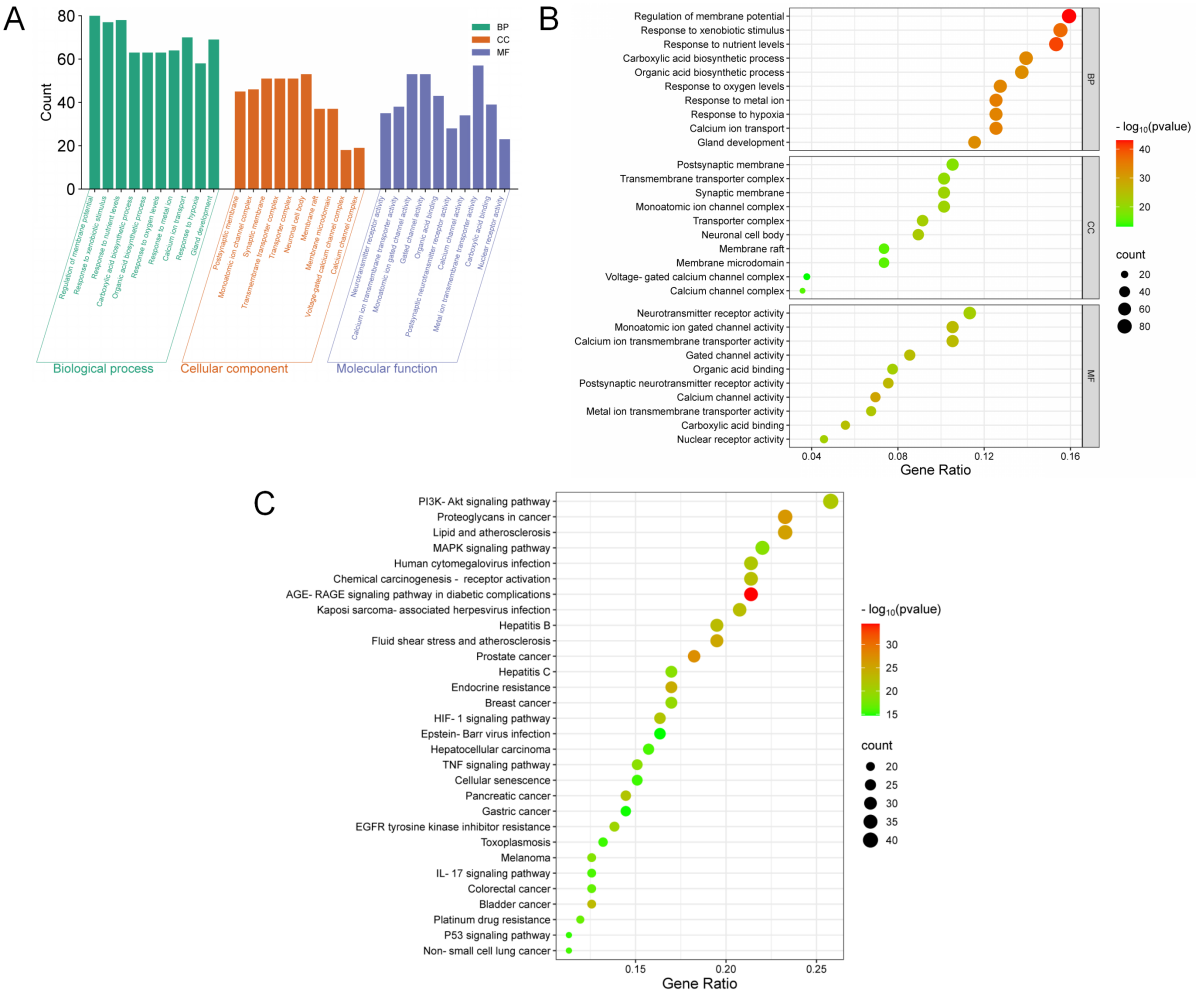
Results of GO and KEGG analysis. **(A)** Bar plot of GO enrichment analysis; **(B)** Bubble plot of GO enrichment analysis; **(C)** Bubble plot of KEGG enrichment analysis.

The 165 intersecting genes were analyzed for GO enrichment with significance thresholds set at *p* < 0.05 and *q* < 0.05. This analysis identified 3,002 potential therapeutic targets for YKD, categorized into 2,708 entries for Biological Processes (BP), 85 entries for Cellular Components (CC), and 209 entries for Molecular Functions (MF). The BPs associated with these potential therapeutic targets included responses to xenobiotic stimuli, epithelial cell proliferation, responses to nutrient levels, and responses to peptide hormones. The GO enrichment analysis prioritized the top 10 entries based on their q-values, ranging from smallest to largest, with the results summarized in Figures 5A,B.

KEGG pathway analysis was conducted employing R and the bioinformatics platform available (see Footnote 7). A total of 186 pathways were identified as enriched with common targets. These pathways were then ranked by their *q*-values, from smallest to largest. From this ranking, 30 pathways were selected for the creation of a KEGG pathway bubble map, as shown in Table 1. In this map, the size of each bubble represents the number of associated genes, and the color intensity reflects the level of significance in gene enrichment. The primary signaling pathways identified include the PI3K-Akt signaling pathway, the lipid and atherosclerosis signaling pathway, the MAPK signaling pathway, and endocrine resistance, as depicted in Figure 5C.

**Table 1:** Top 30 PCOS-related KEGG pathways.

KEGG	Term	Qvalue	Count
Hsa04151	PI3K-Akt signaling pathway	1.92E-21	41
Hsa05205	Proteoglycans in cancer	6.01E-26	37
Hsa05417	Lipid and atherosclerosis	3.93E-25	37
Hhsa04010	MAPK signaling pathway	1.17E-18	35
Hsa04933	AGE-RAGE signaling pathway in diabetic complications	2.72E-33	34
Hsa05207	Chemical carcinogenesis - receptor activation	2.46E-22	34
Hsa05163	Human cytomegalovirus infection	1.41E-21	34
Hsa05167	Kaposi sarcoma-associated herpesvirus infection	1.86E-22	33
Hsa05418	Fluid shear stress and atherosclerosis	1.43E-24	31
Hsa05161	Hepatitis B	1.29E-22	31
Hsa05215	Prostate cancer	1.42E-26	29
Hsa01522	Endocrine resistance	4.58E-24	27
Hsa05224	Breast cancer	1.93E-19	27
Hsa05160	Hepatitis C	1.17E-18	27
Hsa04066	HIF-1 signaling pathway	8.72E-22	26
Hsa05169	Epstein–Barr virus infection	4.90E-15	26
Hsa05225	Hepatocellular carcinoma	6.80E-16	25
Hsa04668	TNF signaling pathway	8.92E-19	24
Hsa04218	Cellular senescence	1.20E-15	24
Hsa05212	Pancreatic cancer	8.72E-22	23
Hsa05226	Gastric cancer	4.34E-15	23
Hsa01521	EGFR tyrosine kinase inhibitor resistance	4.79E-20	22
Hsa05145	Toxoplasmosis	1.20E-15	21
Hsa01522	Endocrine resistance	4.58E-24	27
Hsa05218	Melanoma	2.43E-18	20
Hsa05210	Colorectal cancer	1.05E-16	20
Hsa04657	IL-17 signaling pathway	6.53E-16	20
Hsa01524	Platinum drug resistance	7.12E-17	19
Hsa05223	Non-small cell lung cancer	9.61E-16	18
Hsa04115	P53 signaling pathway	1.89E-15	18

### 3.6 Construction of the YKD-PCOS-KEGG pathways network map

**Figure 6:**
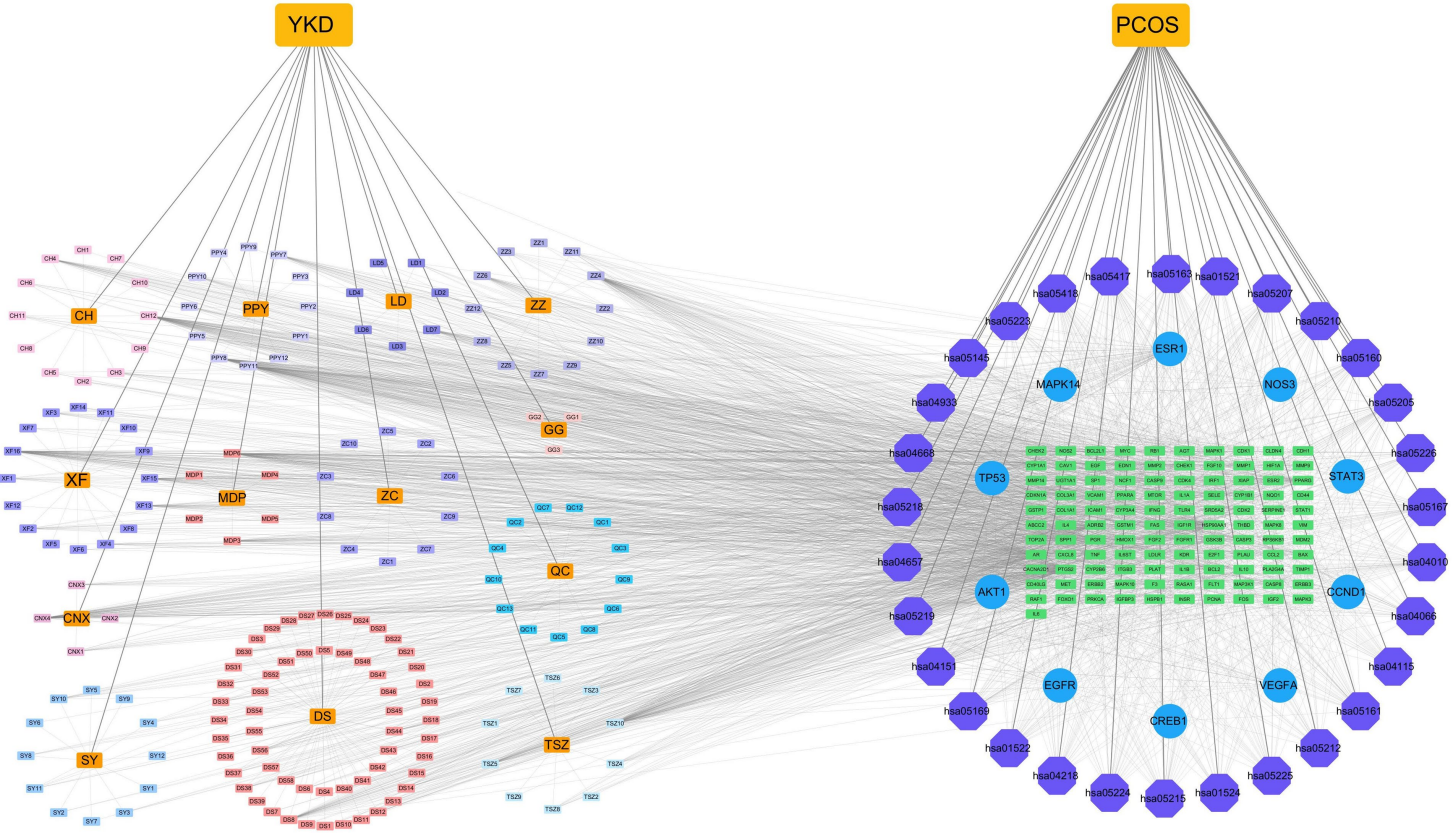
Network diagram of “YKD-PCOS-KEGG.” Pathway. Note: orange rectangular nodes represent drugs, rectangular nodes represent active ingredients, purple octagonal nodes represent KEGG pathways, blue circular nodes represent core targets and green rectangular nodes represent intersecting targets.

The active ingredients of YKD, along with the intersecting targets, the 10 most central targets of YKD-PCOS, and 30 KEGG pathways, were used to construct a network diagram with Cytoscape 3.10.1 (Figure 6).

### 3.7 Molecular docking results

**Figure 7:**
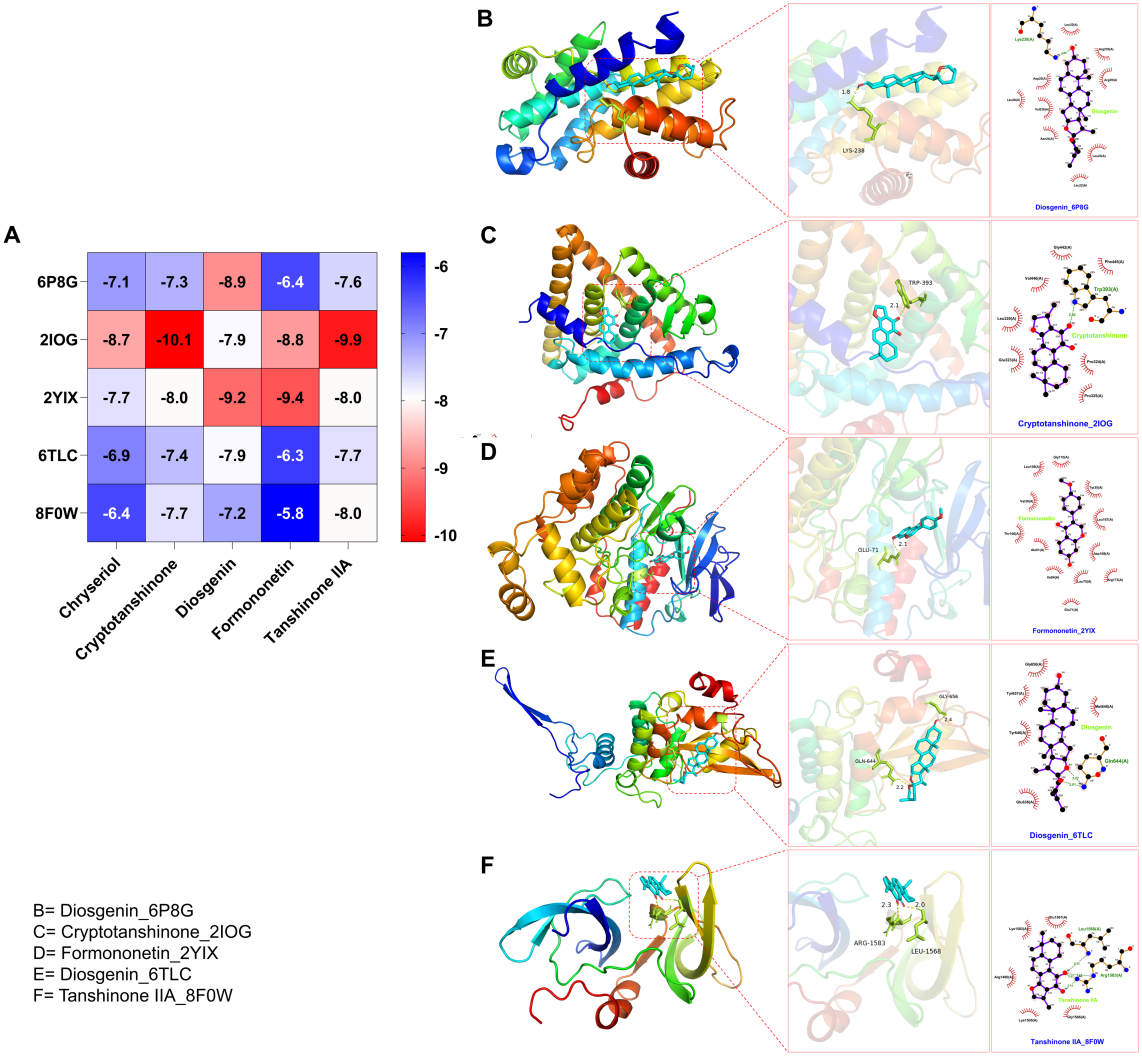
**(A)** Heat map of molecular docking score. Docking results of Binding energy(kcal·mol^−1^) of key targets and active compounds of herbs in 2D and 3D images: **(B)** Diosgenin_6P8G; **(C)** Cryptotanshinone_2IOG; **(D)** Formononetin_2YIX; **(E)** Diosgenin_6TLC; **(F)** Tanshinone IIA_8F0W.

After identifying the core genes using Cytoscape, target proteins (6P8G, 2IOG, 2YIX, 6TLC, 8F0W) were retrieved from the PDB database based on the corresponding genes associated with the active ingredients. The core active ingredients—Chryseriol, Cryptotanshinone, Diosgenin, Formononetin, and Tanshinone IIA—were used as ligand molecules. The spatial coordinates for docking, as well as the details of the targets and compounds, are provided in Table 2. The ligands and receptors were docked separately, and the binding energy values are illustrated in Figure 7A. The results indicated that Cryptotanshinone_2IOG had the lowest binding energy of −10.2 kcal·mol^−1^. Based on the principle of lowest energy, the top candidate from each group was selected for visualization, analysis, and MD. Figures 7B–F present the 3D and 2D plots of the core components and their corresponding core targets visualized after molecular docking.

**Table 2:** Details of targets and compounds for molecular docking.

Target	PDB ID	Center coordinate	Compound	Chemical structure
CCND1	6P8G	0.117, −11.577, −51.152	Chryseriol	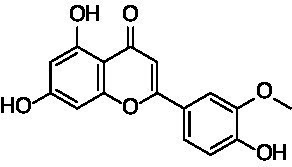
ESR1	2IOG	22.508, 5.063, 19.976	Cryptotanshinone	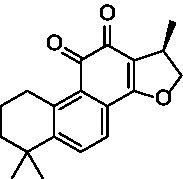
MAPK14	2YIX	1.936, 19.645, 37.344	Diosgenin	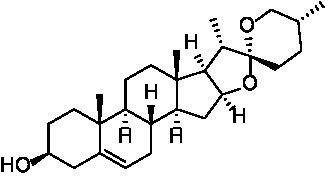
STAT3	6TLC	−26.559, 23.797, −2.593	Formononetin	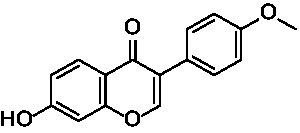
TP53	8F0W	−15.431, −6.114, 6.048	Tanshinone IIA	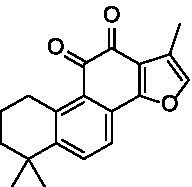

### 3.8 MD results

**Figure 8:**
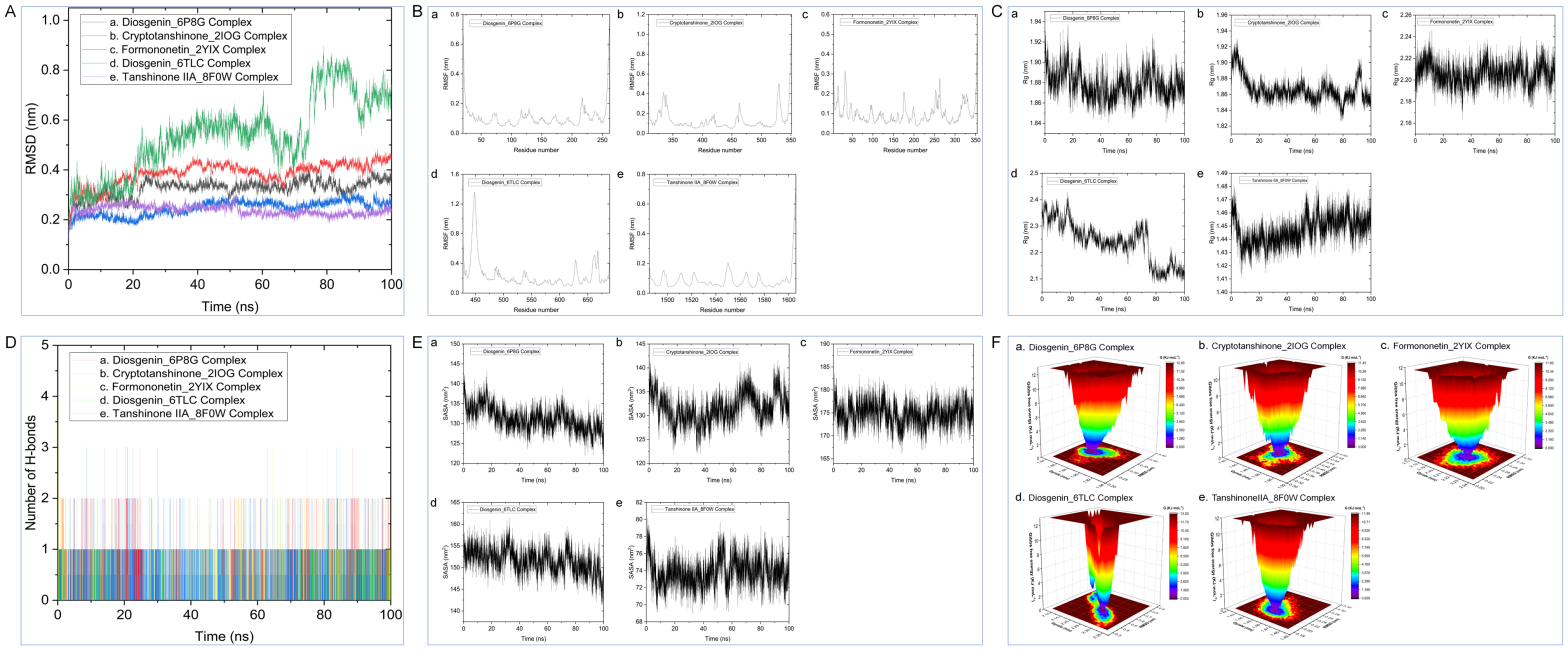
**(A)** RMSD; **(B)** RMSF; **(C)** Rg; **(D)** SASA; **(E)** NHB; **(F)** PCA plots during of molecular dynamics simulation.

Based on the molecular docking results, the following protein-ligand complex systems were analyzed using 100 ns MD simulations: Diosgenin_6P8G, Cryptotanshinone_2IOG, Formononetin_2YIX, Diosgenin_6TLC, and Tanshinone IIA_8F0W. After performing the 100 ns MD simulations, trajectory files were generated, and the stability of these complex systems was assessed through various analyses, including RMSD, RMSF, Rg, SASA, H-bond changes, and PCA. The detailed results of these analyses are presented in Figure 8.

## 4 Discussion

PCOS is a prevalent gynecologic endocrine disorder that presents significant challenges for both families and society. With declining fertility rates and a trend toward delayed marriage, PCOS is becoming a major global public health concern (19). The disorder can lead to insulin resistance, hyperlipidemia, and metabolic syndrome (MS), which in turn increase the risk of glucose intolerance, type 2 diabetes mellitus, cardiovascular disease, and neoplasms in women (20, 21). Additionally, PCOS is associated with dyslipidemia, as well as emotional and psychosexual disorders, posing a serious threat to overall health and well-being. TCM, which has undergone extensive development over thousands of years, has had a profound and far-reaching impact both within China and globally. TCM offers distinct advantages in managing various illnesses, particularly chronic and challenging conditions (22, 23). Currently, our research group has not conducted in-depth basic research beyond evaluating clinical efficacy and animal experiments. Given the multi-component and multi-target nature of TCM, a comprehensive research approach is required to analyze the mechanisms of YKD in treating PCOS. Consequently, we employed network pharmacology combined with molecular docking and MD simulations to explore the mechanisms of YKD’s potential active components in the treatment of PCOS.

Following a network pharmacological analysis, this study identified 139 probable active components and proposed that YKD may exert its therapeutic effects on PCOS through the actions of Chryseriol, Cryptotanshinone, Diosgenin, Formononetin, and Tanshinone IIA. Chryseriol, sourced from Rhizoma Cyperi Preparata, possesses a range of biological activities, including antioxidant, anti-inflammatory, and hormone-regulating effects, making it a valuable candidate for studying and treating endocrine system diseases (24). Notably, Chryseriol has demonstrated phytoestrogen-like activity, which has garnered interest in the context of gynecological disorders such as menstrual irregularities and PCOS (25). Additionally, Cryptotanshinone and Tanshinone IIA are derived from Radix Salviae liguliobae.

Cryptotanshinone, a diterpene quinone compound, has been shown to modulate estrogen receptors and potentially influence estrogen activity (26). This has made it a focal point in studies related to breast cancer, endometriosis, and other estrogen-related diseases. Besides, research indicates that Cryptotanshinone may impact insulin signaling and glucose metabolism, generating interest in its potential applications for diabetes and metabolic disease research (27). Tanshinone IIA inhibits estrogen synthesis and promotes luteinizing hormone production, which may help restore the balance between estrogen and luteinizing hormone, thereby improving the PCOS (28). Furthermore, Tanshinone IIA enhances ovarian function and promotes oocyte development in mice, contributing to the restoration of normal ovulatory cycles (29). Diosgenin, derived from Rhizoma Dioscoreae, has been found to exhibit phytoestrogen-like effects in menopausal syndrome and estrogen-related disorders. It may help relieve symptoms of menopausal syndrome and ameliorate issues associated with estrogen deficiency (30). Moreover, Diosgenin has been investigated for its potential in treating PCOS, as it may modulate ovarian function and androgen levels, thereby improving symptoms and signs in PCOS patients (31). Formononetin, derived from Pueraria Mirifica, has demonstrated in some animal and *in vitro* studies the ability to mimic the biological activity of estrogen, modulate the insulin signaling pathway, ameliorate insulin resistance, and regulate the secretion of ovarian hormones. This suggests that Formononetin plays a modulating role in PCOS patients with abnormal estrogen levels (32, 33). Notably, Formononetin possesses antioxidant, anti-inflammatory, and anti-metabolic syndrome properties, which may positively affect symptoms and metabolic abnormalities in PCOS patients (34). The study of the PPI interaction network and CytoNCA findings revealed that the primary targets of YKD for the treatment of PCOS are CCND1, ESR1, MAPK14, STAT3, and TP53. Notably, the CCND1 protein plays a critical role in regulating biological processes such as cell growth, proliferation, and differentiation. It is also involved in regulating cell regeneration and repair processes in normal tissues, which is essential for maintaining the physiological functions of these tissues and organs (35). In patients with PCOS, ovarian follicle growth is often impaired, primarily due to an imbalance between the proliferation and apoptosis of ovarian granulosa cells. CCND1 influences the normal growth and maturation of follicles by controlling cell proliferation and differentiation. It promotes cell proliferation by interacting with CDK4/6, which facilitates the phosphorylation of the Rb protein and releases the E2F transcription factor, thereby enabling cells to transition from the G1 phase to the S phase (36). Estrogen receptor alpha (ERα), encoded by the ESR1 gene, plays a crucial regulatory role in ovarian tissues. It is involved in regulating growth, differentiation, and hormone sensitivity. Research on PCOS suggests that the ESR1 gene and the estrogen receptor it encodes may significantly contribute to the pathogenesis of PCOS (37). Polymorphisms in the ESR1 gene and abnormal expression of the estrogen receptor have been associated with the pathophysiological characteristics of PCOS patients. Certain polymorphic variants in the ESR1 gene have been identified in some PCOS patients, potentially affecting estrogen receptor function and estrogen signaling (38). Additionally, some studies have indicated that the expression levels of estrogen receptors in the ovarian tissues of PCOS patients may be altered, potentially contributing to impaired follicular development and abnormal ovulation. Beyond directly affecting the biological functions of ovarian tissues, the ESR1 gene and its encoded estrogen receptor may also regulate the balance of estrogen and other hormones by influencing endocrine system function, thereby impacting the endocrine metabolic status of PCOS patients (39). MAPK14, also known as p38 MAP kinase, is a member of the mitogen-activated protein kinase (MAPK) family, which regulates various biological processes, including cell proliferation, differentiation, apoptosis, and inflammatory responses. Studies related to PCOS suggest that MAPK14 may be involved in the disease’s pathogenesis (40). Research has shown that in PCOS, a common gynecological condition caused by hormonal imbalance and abnormal proliferation of ovarian tissues—there is often increased activity and expression of MAPK14 in the ovarian tissues of affected patients (41). STAT3, a crucial transcription factor, plays a significant role in regulating various cellular processes, including cell proliferation, differentiation, apoptosis, and inflammatory responses (42). The study revealed a notable increase in both the activity and expression levels of STAT3 in the ovarian tissues of rats with PCOS (43, 44). This increase may lead to excessive cell proliferation and impaired follicular development, resulting in disrupted ovulation and the formation of cystic ovaries. Overexpression of STAT3 contributes to ovarian dysfunction and insulin resistance in mice, further exacerbating pathological changes (45). The primary functions of TP53 include regulating the cell cycle, inducing apoptosis, and maintaining genome stability (46). Research has shown that TP53 expression is significantly reduced in the ovarian tissues of individuals with PCOS. This reduction may lead to increased cell growth, decreased apoptosis, and impaired formation of ovarian follicles (47). Lower TP53 activity can disrupt the cell cycle and diminish the repair of DNA damage, resulting in abnormal ovarian tissue growth and the development of polycystic ovaries. In addition, TP53 is involved in the pathogenesis of PCOS by affecting the inflammatory response and metabolic regulation of ovarian tissues, among other factors (48).

Furthermore, to provide a clearer understanding of the current research on these components and their key mechanisms of action, we have summarized the relationships between five active compounds and their respective targets, based on findings from previously published literature.

Previous research demonstrates that Chrysoeriol effectively alleviates skin inflammation by modulating STAT3 transcription, leading to reduced mRNA levels of the pro-inflammatory cytokines IL-6, IL-1β, and TNF-*α* (49). Chrysoeriol also inhibits the excessive proliferation of rheumatoid arthritis fibroblast-like synoviocytes (RA-FLS) by preventing the activation and phosphorylation of STAT3 (Tyr705), lowering STAT3 nuclear levels, and downregulating the expression of Bcl-2 and Mcl-1, both of which are regulated by STAT3 (50). Additionally, studies have indicated that the combined administration of Cryptotanshinone and Temozolomide has a significantly stronger inhibitory effect on cell proliferation than when each drug is used individually. Western blot analysis confirmed that the combination therapy markedly reduced STAT3 expression in glioblastoma multiforme cells compared to Temozolomide alone (51). Cryptotanshinone exhibits lethal effects on ER-positive breast cancer cells by modulating their proliferation and migration, primarily through the inhibition of ESR1 (52). Additionally, diosgenin directly inhibits the phosphorylation of STAT3 and its transcriptional regulation of NPC1L1 expression, leading to a significant reduction in NPC1L1 levels. This downregulation hinders intestinal cholesterol absorption, thereby preventing the formation of cholesterol gallstones (53). In food allergy responses, elevated levels of IgE and hyperresponsive mast cells and basophils are critical factors. Validation through qPCR demonstrates that Formononetin suppresses IgE-mediated mast cell activation by upregulating TP53 gene expression and downregulating the expression of STAT3 and CCND1, ultimately reducing the severity of food allergies (54). The proposed anti-allodynic and anti-neuroinflammatory effects of sodium tanshinone IIA sulfonate (STS), a derivative of tanshinone IIA, have been observed in neuropathic pain models, likely involving the miR-125b-5p/STAT3 axis (55). In conclusion, the clear correlation between these components and their specific targets underscores the significance of our study on YKD, warranting further investigation.

The results of GO analysis for relevant targets indicate that YKD demonstrates efficacy in PCOS by influencing various biological processes, cellular components, and molecular functions. Among these aspects, biological processes showed the highest level of enrichment, representing 90.21% of the GO analysis results. Key biological processes identified include the response to xenobiotic stimuli, epithelial cell proliferation, response to nutrient levels, and response to peptide hormones. GO analysis is instrumental in elucidating the molecular mechanisms underlying PCOS and enhancing our understanding of its onset and progression. By examining significantly enriched GO terms, researchers can pinpoint critical biological processes, molecular functions, and cellular components involved in PCOS. This information supports further functional studies, facilitates the identification of potential therapeutic targets, and aids in the development of novel treatment strategies.

The KEGG analysis indicates that YKD treatment for PCOS may influence several signaling pathways, including the PI3K-Akt signaling pathway, lipid and atherosclerosis signaling pathway, MAPK signaling pathway, and endocrine resistance pathway. In PCOS patients, the PI3K-Akt signaling pathway may be abnormally activated or dysfunctional, leading to metabolic disorders, ovarian hormonal imbalances, and the formation of ovarian polycysts (56). Specifically, PI3K (phosphatidylinositol 3-kinase) is the initiating enzyme of the PI3K-Akt signaling pathway, and its primary role is to transmit external growth factor signals into the cell. In patients with PCOS, the expression and activity of PI3K are increased, leading to over-activation of the PI3K-Akt signaling pathway. Akt (protein kinase B), a key component of this pathway, regulates several downstream signaling molecules, including mTOR, GSK3β, and FOXO, after phosphorylation. In PCOS patients, elevated phosphorylation levels of Akt exacerbate the activity of the PI3K-Akt signaling pathway. Excessive activation of this pathway can impact various biological processes within ovarian tissue, such as follicular development, ovarian hormone synthesis and secretion, and metabolic regulation (57). These effects contribute to features such as ovarian polycysticity, abnormal estrogen levels, and insulin resistance in PCOS patients. PCOS is a common endocrine disorder frequently associated with metabolic abnormalities, including insulin resistance, high cholesterol, and hypertriglyceridemia. These metabolic issues can increase the risk of atherosclerosis. There is a complex relationship between lipid metabolism, atherosclerotic signaling pathways, and PCOS. Abnormalities in lipid metabolism are common among PCOS patients (58). Due to insulin resistance, patients with PCOS often exhibit dyslipidemia, such as elevated cholesterol, high triacylglycerols, low-density lipoprotein (LDL), and very low-density lipoprotein (VLDL) (59). These lipid imbalances can disrupt metabolism, leading to lipid accumulation in blood vessels and potential atherosclerotic plaque formation. Atherosclerosis is a chronic inflammatory disease involving multiple inflammatory cells and factors (60). In PCOS patients, chronic low-grade inflammation, associated with metabolic abnormalities and abnormal androgen levels, may contribute to atherosclerosis development (61). Consequently, weight gain and abdominal obesity in PCOS patients can trigger an inflammatory response and insulin resistance, further increasing the risk of atherosclerosis (62). In PCOS patients, the imbalance between estrogen and androgen levels leads to abnormal activation of the MAPK signaling pathway in ovarian and other related cells. This pathway is involved in regulating physiological processes such as follicular development, androgen synthesis, and the proliferation and apoptosis of ovarian cells (63). Consequently, this can lead to abnormal ovarian function and cyst formation, contributing to PCOS development. Moreover, the chronic low-grade inflammatory state in PCOS patients is linked to the MAPK signaling pathway, which plays a significant role in the inflammatory response. Activated MAPK signaling can promote the release of inflammatory factors, exacerbating the inflammatory response and affecting the body’s metabolic and endocrine balance. These changes are closely associated with the pathogenesis of PCOS. Endocrine resistance, a common phenomenon where the body’s response to endocrine therapy weakens or fails, reduces the effectiveness of treatments and complicates disease management (64). PCOS patients often exhibit insulin resistance and elevated androgen levels, which can lead to resistance to certain endocrine therapies (65). In summary, multiple intricate signaling pathways play crucial roles in the pathological progression of PCOS, including those involved in cell survival, proliferation, differentiation, metabolic regulation, inflammatory response, and endocrine regulation. A comprehensive examination and understanding of these pathways will enhance the treatment strategies for PCOS.

Combining the perspectives of semi-flexible docking and energy stabilization, the visualization revealed that Chryseriol binds to CCND1 (PDB ID: 6P8G) via residue LYS-238, Cryptotanshinone binds to ESR1 (PDB ID: 2IOG) via residue TPR-392, Diosgenin binds to MAPK14 (PDB ID: 2YIX) via residue GLU-71, Formononetin binds to STAT3 (PDB ID: 6TLC) via residues GLN-644 and GLY-656, and Tanshinone IIA binds to STAT3 (PDB ID: 6TLC) via residues LEU-1568 and GLY-656. The binding energies for these interactions were − 8.9 kcal·mol^−1^, −10.1 kcal·mol^−1^, −9.4 kcal·mol^−1^, −7.9 kcal·mol^−1^, and − 8.0 kcal·mol^−1^, respectively. The binding energies were all <−7.5 kcal·mol^−1^, indicating strong affinity and stability. These findings suggest that these compounds may play significant roles in YKD’s pharmacological effects. However, the results of molecular docking might not always reflect the most accurate docking scenarios. To address this, we performed MD simulations on the complex systems. Using Gromacs, we conducted MD simulations on the most favorable conformations obtained from molecular docking. Through various analyses, including Root Mean Square Deviation (RMSD), Root Mean Square Fluctuation (RMSF), Radius of Gyration (Rg), Solvent Accessible Surface Area (SASA), Number of H-bonds (NHB), and Principal Component Analysis (PCA), we obtained further evidence supporting the stability and strong binding of Diosgenin with CCND1 (6P8G), Cryptotanshinone with ESR1 (2IOG), Formononetin with MAPK14 (2YIX), Diosgenin with STAT3 (6TLC), and Tanshinone IIA with TP53 (8F0W).

## 5 Summary

This study comprehensively investigated the potential of YKD for treating PCOS through network pharmacology, molecular docking, and MD techniques. The results suggest that YKD may exert its therapeutic effects on PCOS through key components such as Chryseriol, Cryptotanshinone, Formononetin, Tanshinone IIA, and Diosgenin. The MD data revealed significant associations between core genes and these components, reinforcing YKD’s therapeutic impact on PCOS. However, further clinical studies and experimental validation are necessary to fully elucidate YKD’s mechanisms in PCOS treatment. Overall, our findings validate the reliability of our molecular docking results and highlight YKD’s potential as a promising treatment for PCOS.

## Data Availability

The original contributions presented in the study are included in the article/supplementary material, further inquiries can be directed to the corresponding author.

## Footnotes

^1^http://lsp.nwu.edu.cn/tcmsp

^2^https://www.genecards.org/

^3^https://www.omim.org/

^4^https://www.pharmgkb.org/

^5^https://www.drugbank.ca/

^6^https://cn.string-db.org/

^7^https://www.bioinformatics.com.cn/

^8^https://www.uniprot.org/

^9^https://www.rcsb.org/
